# Neuroendocrine tumours of the female genital tract: a case-based imaging review with pathological correlation

**DOI:** 10.1007/s13244-014-0378-5

**Published:** 2015-01-16

**Authors:** João Lopes Dias, Teresa Margarida Cunha, Filipe Veloso Gomes, Catarina Callé, Ana Félix

**Affiliations:** 1 Department of Radiology, Hospital de São José, Centro Hospitalar de Lisboa Central, Lisbon, Portugal; 2 Department of Radiology, Instituto Português de Oncologia de Lisboa Francisco Gentil, Lisbon, Portugal; 3 Department of Radiology, Centro Hospitalar do Algarve, Algarve, Portugal

**Keywords:** Poorly differentiated neuroendocrine carcinomas (NECs), Well-differentiated neuroendocrine tumours (NETs), Small cell carcinoma, Large cell neuroendocrine carcinoma, Carcinoid

## Abstract

**Background:**

Both primary and secondary gynaecological neuroendocrine (NE) tumours are uncommon, and the literature is scarce concerning their imaging features.

**Methods:**

This article reviews the epidemiological, clinical and imaging features with pathological correlation of gynaecological NE tumours.

**Results:**

The clinical features of gynaecological NE tumours are non-specific and depend on the organ of origin and on the extension and aggressiveness of the disease. The imaging approach to these tumours is similar to that for other histological types and the Revised International Federation of Gynecology and Obstetrics (FIGO) Staging System also applies to NE tumours. Neuroendocrine tumours were recently divided into two groups: poorly differentiated neuroendocrine carcinomas (NECs) and well-differentiated neuroendocrine tumours (NETs). NECs include small cell carcinoma and large cell neuroendocrine carcinoma, while NETs account for typical and atypical carcinoids. Cervical small cell carcinoma and ovarian carcinoid are the most common gynaecological NE tumours. The former typically behaves aggressively; the latter usually behaves in a benign fashion and tends to be confined to the organ.

**Conclusion:**

While dealing with ovarian carcinoids, extra-ovarian extension, bilaterality and multinodularity raise the suspicion of metastatic disease. NE tumours of the endometrium and other gynaecological locations are very rare.

***Teaching Points*:**

• *Primary or secondary neurondocrine (NE) tumours of the female genital tract are rare.*

• *Cervical small cell carcinoma and ovarian carcinoids are the most common gynaecological NE tumours.*

• *Cervical small cell carcinomas usually behave aggressively.*

• *Ovarian carcinoids tend to behave in a benign fashion.*

• *The imaging approach to gynaecological NE tumours and other histological types is similar*.

## Introduction

Neuroendocrine tumours comprise a set of neoplasms that arises from the diffuse neuroendocrine cell system. These tumours are more commonly identified in the gastrointestinal tract, pancreas, lung and thymus. Gynaecological NE tumours are uncommon, either as primary or secondary tumours [1].

Recently, a simplified terminology has been proposed, dividing NE tumours in two groups: poorly differentiated neuroendocrine carcinomas (NECs) and well-differentiated neuroendocrine tumours (NETs). NECs include small cell carcinoma and large cell neuroendocrine carcinoma, while NETs account for typical and atypical carcinoids [1–4].

The most prevalent gynaecological NE tumours are cervical small cell carcinoma and ovarian carcinoids. Elsewhere in the female genital tract, NECs or NETs are rare or nonexistent [5]. The clinical features of gynaecological NE tumours generally depend on the organ of origin and are non-specific. However, some patients may present with paraneoplastic syndromes owing to the production of several peptides and hormones. The absence of endocrine clinical syndromes in most patients with NE tumours is possibly related to the release of insufficient amounts or inactive hormones [6].

The purpose of this article is to review the epidemiological, clinical and imaging features with pathological correlation of primary gynaecological NE tumours, emphasising the importance of differentiating them from metastatic disease and other carcinomas with an NE component.

## General histological considerations

For the classification of NE differentiation, tumour cells have to express at least two NE markers such as chromogranin A, synaptophysin or neuron-specific enolase. A great variety of other peptides and hormones may be found, including calcitonin, gastrin, serotonin, substance P, vasoactive intestinal peptide, pancreatic polypeptide, somatostatin and adrenocorticotrophic hormone [1, 3].

Some cancers display a combination of NE and non-NE features, usually either glandular or squamous components. The amount of NE component within a non-NE carcinoma may range from a single NE cell to a well-identifiable NE tumour cell population. In this set, two terms are widely accepted: mixed exocrine-endocrine carcinoma (MEEC) and (adeno)carcinoma with (focal) NE differentiation. The finding of a focal non-NE differentiation in almost pure NE tumours is less common [7].

According to the World Health Organisation (WHO) classification of NE tumours, the diagnosis of a mixed exocrine-endocrine tumour should take into account at least two major diagnostic parameters: an extension of at least 30 % for each component and the recognition of structural NE features such as well-differentiated organoid or solid/diffuse growth patterns. In fact, no reasonable explanation is provided for the limit of 30 %, and consequently morphological criteria should also be considered [7, 8].

Carcinomas with focal NE differentiation have been identified in several non-NE tumours of the gynaecological tract, such as endometrial endometrioid adenocarcinomas, endocervical adenocarcinomas and adenosquamous carcinomas, and even ovarian surface epithelial neoplasms of mucinous, endometrioid and serous types. These tumours display less than one-third of NE cells and a non-NE growth pattern, and the finding of these NE cells has not been shown to affect outcome [5, 7].

Few data are currently at our disposal regarding the prognosis of mixed exocrine-endocrine carcinoma (MEEC) in comparison to pure NE or non-NE tumours. However, the therapeutic schemes are different and the accurate histological diagnosis is mandatory.

The diagnosis of an NE tumour may be challenging. The differentiation of small cell NE carcinomas from poorly differentiated squamous cell carcinoma (SCC) with NE features may be especially difficult. Sometimes, and particularly for cervical cancers, the recognition of the NE component is only possible after the hysterectomy, since the tissue obtained on cervical biopsy may be insufficient, leading to the diagnosis of poorly differentiated cervical cancer.

## General imaging considerations

Due to the rarity of these tumours, data in the literature concerning their imaging features are scarce. We know that small cell carcinoma and large cell NE carcinoma tend to behave aggressively, in contrast to carcinoids, which are typically confined to the organ. Nevertheless, these are non-specific features and the available data regarding these extremely rare tumours are limited and discourage scientific generalisations. Consequently, no reliable guidelines, for either diagnosis or treatment, are established. For staging purposes, physicians and radiologists should consider the currently used Revised International Federation of Gynecology and Obstetrics (FIGO) Staging System [9–11].

The imaging approach to gynaecological NE tumours is similar to that for other histological types. For the cervix, uterus and adnexa, ultrasound (US) using both transabdominal and transvaginal probes plays a critical role in the initial diagnosis. The use of computed tomography (CT) or positron emission tomography-computed tomography (PET-CT) is established in cervical and endometrial tumours for detection of nodal and distant metastases, which may be particularly useful for poorly differentiated NE carcinomas, typically invasive and metastatic. In ovarian cancers, CT is also indicated to evaluate the extent of disease and detect nonresectable metastases. Magnetic resonance imaging (MRI) may be used for characterising indeterminate adnexal masses and for pre-treatment staging purposes, essentially in cervical and endometrial tumours. MRI may replace CT in the staging of adnexal tumours in young females, pregnancy, renal insufficiency and allergy to iodinated contrast [10, 12, 13] (Table 1).

**Table 1:** Histological classification of NE tumours. Table created by the authors based on the literature review [1–4, 7]

Poorly differentiated neuroendocrine carcinomas (NECs)	Small cell carcinoma
	Large cell neuroendocrine carcinoma
Well-differentiated neuroendocrine tumours (NETs)	Typical carcinoid
	Atypical carcinoid
Combination of NE and non-NE features	Mixed exocrine-endocrine carcinoma (MEEC) (adeno) carcinoma with (focal) NE differentiation

NE tumours of the vagina and vulva are much rarer. US plays a limited role in the diagnosis, but CT and MRI may be used for staging purposes. MRI is primarily used for local staging of vaginal tumours and should be performed after vaginal filling with gel [11].

Particularly for typical and atypical carcinoids (well-differentiated neuroendocrine tumours), an octreotide scan may be used. This is a somatostatin analogue scintigraphy that assesses relevant receptor expression in vivo. Patients with negative octreotide scans should not be considered for somatostatin analogue therapy and typically show better responses to chemotherapy schemes [1].

## Ovary

The most common NE tumours of the ovary are typical carcinoid tumours. Atypical carcinoids and poorly differentiated NE carcinomas (NECs) are very rare.

## Well-differentiated neuroendocrine tumours (NETs)

Primary carcinoid tumours of the ovary are rare and account for less than 5 % of all carcinoid tumours and for less than 0.1 % of all ovarian neoplasms. These are germ cell tumours in origin and classified as teratomas with a predominance of NE features. The great majority is asymptomatic and incidentally found on cross-sectional or ultrasound imaging. The median age of diagnosis is 55 years of age (range from 17 to 83 years), with most of the patients being peri- or postmenopausal [1, 14–17].

These tumours are typically unilateral, slow growing and diagnosed in an early stage. In a 5-decade analysis performed by Modlin et al. [18], 66 % of 113 carcinoid ovarian tumours were localised lesions, while 22 %–31 % presented with distant spread. They may exist in pure forms, predominantly solid with small cysts or as a nodule within a mature teratoma, mucinous cystadenoma or even Brenner tumour [19].

Four distinct histological subtypes are described: insular, trabecular, mucinous and strumal. Ovarian carcinoids contain well-differentiated NE cells and some subtypes are similar to the gastrointestinal carcinoids. They are immunoreactive to at least one neuroendocrine marker, including chromogranin, synaptophysin or Leu-7, and may also be present in peptide hormones such as pancreatic polypeptide, gastrin, vasoactive intestinal peptides and glucagon [14].

Insular carcinoid is the most common type of ovarian carcinoid, typically presenting with a pelvic mass. Near 30 % of patients will have signs and symptoms of carcinoid syndrome, including facial flushing, diarrhoea, bronchospasm and oedema [20]. It happens because serotonin-like substances are directly released into the systemic circulation through the ovarian venous system, bypassing hepatic deactivation. Carcinoid syndrome is less common in the other subtypes (13 % in trabecular and 3.2 % in strumal carcinoids) [16].

Trabecular carcinoid constitutes the second most common ovarian carcinoid tumour and 25 % of patients will present with constipation due to peptide YY production, which inhibits intestinal motility [15, 17]. Some cases of hirsutism are also thought to be related to peptide YY production, which is known to inhibit peripheral steroid-producing cells [14, 19].

Mucinous carcinoids are rare (1.5 %), may be pure or associated with mature teratomas. In these cases, the differential diagnosis must include metastasis from a gastrointestinal tumour and primary mucinous appendiceal carcinoid [1].

We should also consider strumal carcinoids, tumours that contain normal thyroid and carcinoid tissues [21]. Eight per cent of the patients may present with thyroid hormone-related symptoms and some ovarian strumal carcinoids are also reported to cause severe constipation owing to peptide YY production [15, 22].

Primary carcinoid tumours typically behave in a benign fashion, and the prognosis is generally favourable. If the tumour presents with stage I, survival may be greater than 90 %; however, for women with advanced stage disease, the prognosis is poor and the 5-year survival is approximately 33 %. Attending to the more favourable prognosis of ovarian carcinoid tumours, the differentiation of such lesions from other highly malignant ovarian neoplasms is mandatory in order to choose the most appropriate therapeutic options. Particular note should be taken in relation to mucinous carcinoid, which behaves more aggressively and may present with extra-ovarian spread and lymph node involvement [14].

While performing the diagnosis of an ovarian carcinoid, it is important to rule out ovarian metastases, essentially from the gastrointestinal tract. The insular and trabecular subtypes most commonly have metastases in the ovary simulating a primary tumour. Metastatic carcinoid is frequently bilateral, while primary ovarian carcinoids tend to be unilateral [1, 23].

Robboy et al. [24] compared the features of primary insular carcinoids and metastatic midgut carcinoids and concluded that the presence of extra-ovarian tumour, bilaterality, multinodularity, vascular invasion and the absence of teratomatous elements are suggestive of an extra-ovarian origin [5].

## Poorly differentiated neuroendocrine carcinomas (NECs)

Both small cell carcinoma and large cell NE carcinoma of the ovary are highly malignant and aggressive, regardless of the stage [1].

Ovarian small cell carcinoma resembles the pulmonary type and is often associated with common epithelial tumours, typically an endometrioid carcinoma, suggesting an origin from ovarian surface epithelium. Its immune profile includes positivity for neuron-specific enolase and less commonly for chromogranin [1, 5].

Large cell NE carcinomas have been associated with benign and malignant surface epithelial-stromal tumours, and immunohistochemical markers for chromogranin are typically positive [25, 26].

**Fig. 1:**
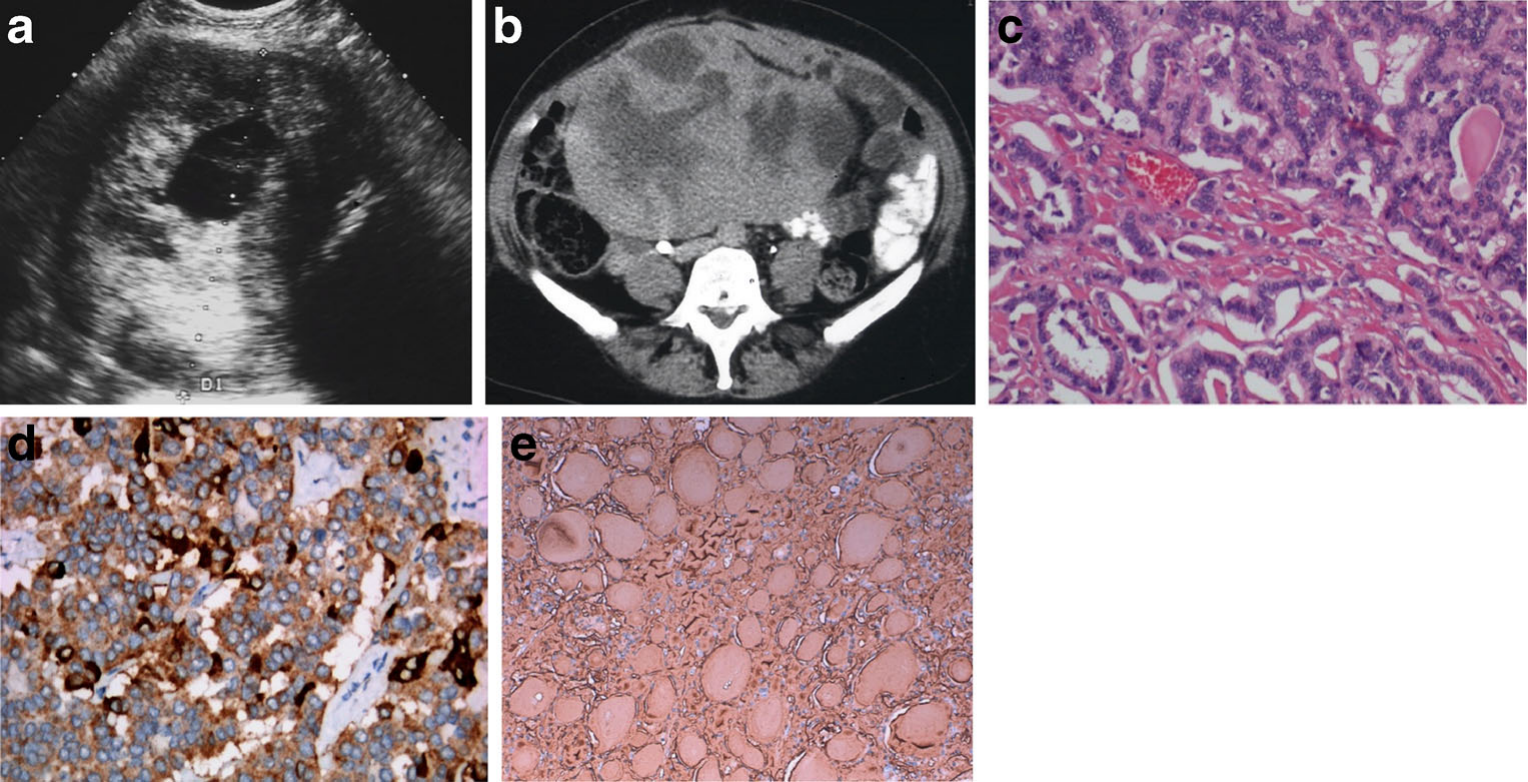
**a**–**e** Struma carcinoid of the right ovary. A 49-year-old female complaining of pelvic and lower abdominal pain. Ultrasound (**a**) and axial enhanced CT image (**b**). Large, heterogeneous, mixed tumour of the ovary, predominantly solid, with necrotic areas and calcifications. The solid portions are moderately echogenic on US and hyperdense on enhanced CT, while cystic areas are anechogenic on US and hypodense on CT. No ascites are found. This tumour is composed of areas of thyroid tissue and carcinoid (H&E), the latter having acinar and trabecular architecture (**c**). The neuroendocrine cells have very uniform nuclei and scarce cytoplasm. The tumour cells show positive cytoplasmatic staining for chromogranine (**d**) and for synaptophysin (not shown). The thyroid area with compact folicular structure presents intense immunoreactivity for thyroglobulin (**e**)

**Fig. 2:**
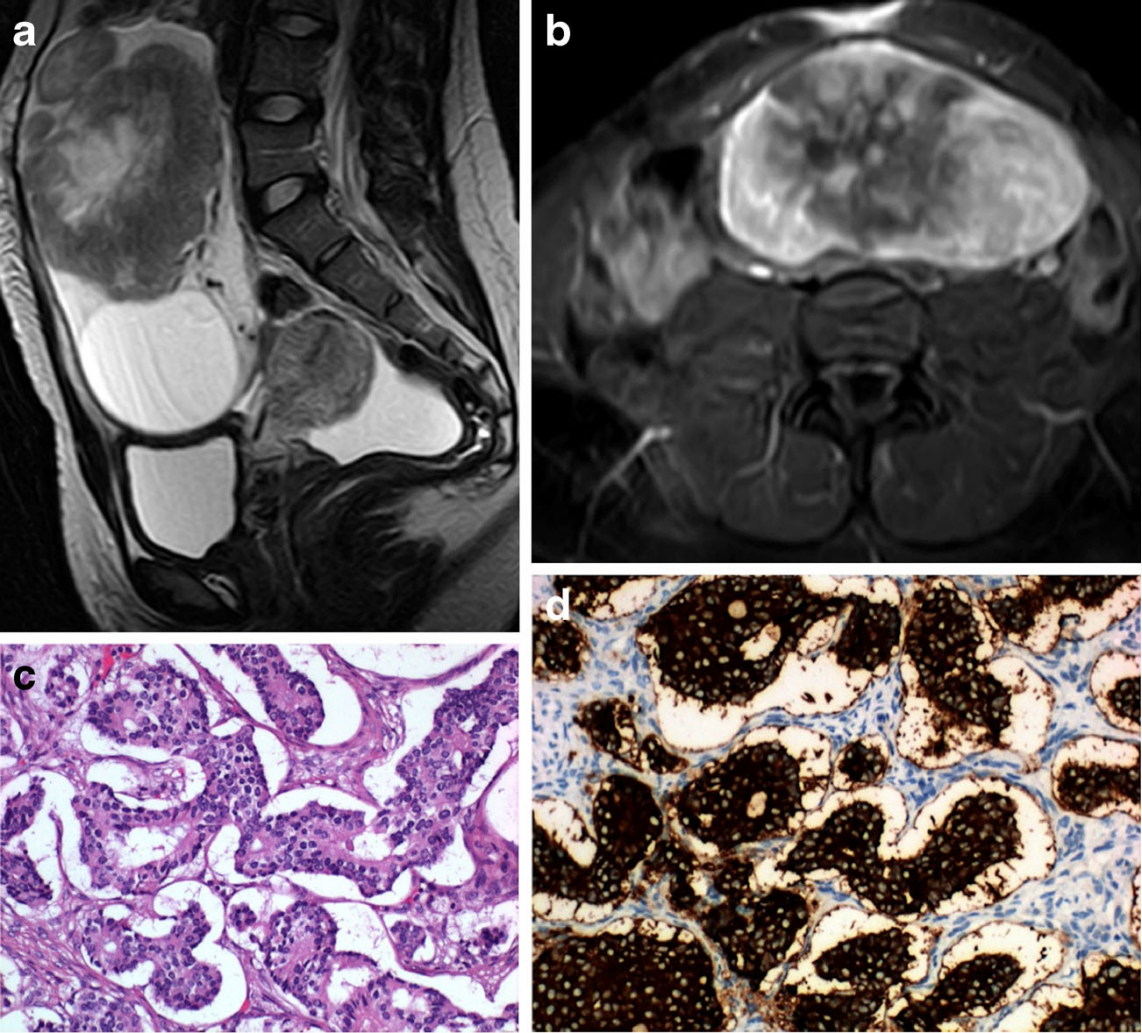
**a**–**d** Metastatic ovarian carcinoid of unknown primary origin in a 23-year-old female complaining of pelvic pain and amenorrhoea. Sagittal T2-weighted MR image (**a**) and axial T1-weighted MR image with fat saturation, after intravenous gadolinium administration (**b**). Large, well-defined solid lesion of the left ovary, cranially and anteriorly located in relation to the uterus. On T2WI, it displays intermediate signal intensity and shows a central high signal region. The lesion shows peripheral, intense contrast enhancement and a large central area of necrosis. Inferiorly to the solid tumour, a large simple cyst is found. A small amount of ascites is found in the cul-de-sac. H&E section of the ovary occupied by a tumour with insular pattern composed by neuroendocrine cells (**c**). These cells have intense cytoplasmatic staining for synaptophysin (**d**) and have small chromogranine-positive granules in the cytoplasm (not shown)

**Fig. 3:**
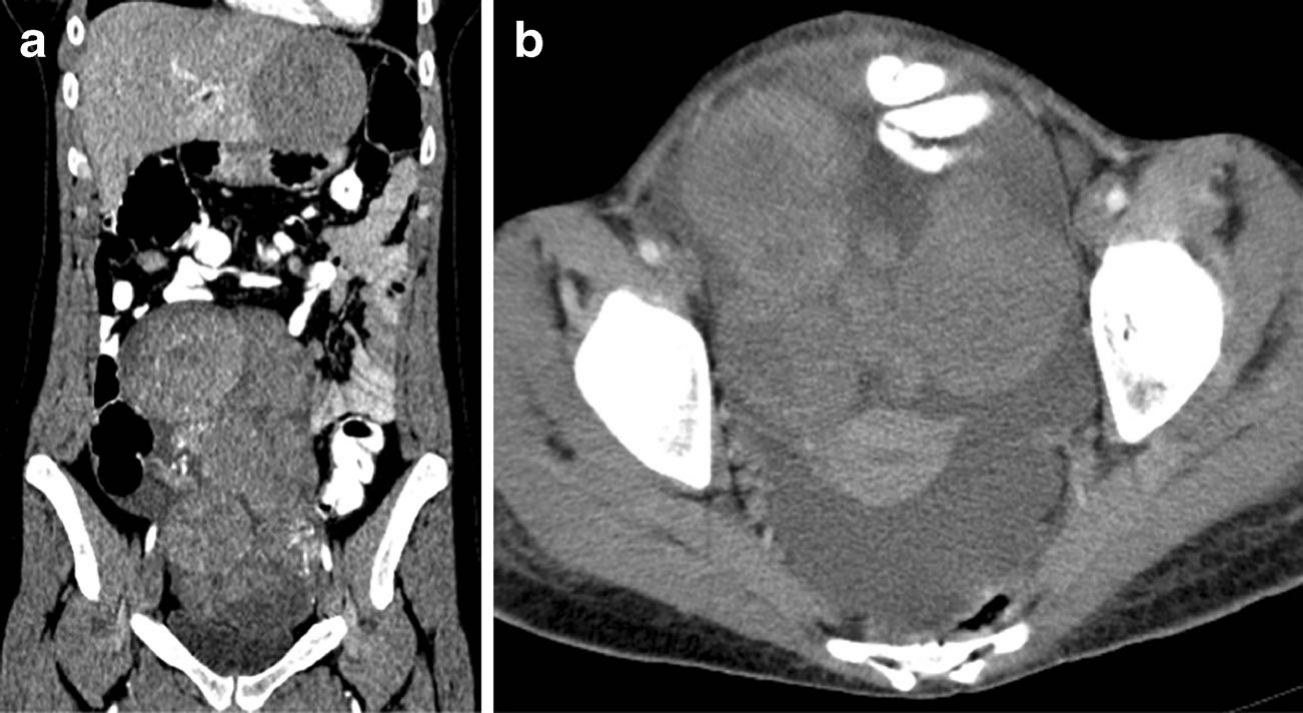
**a**–**b** Metastatic, bilateral ovarian carcinoid, from a primary jejunal tumour in a 28-year-old female complaining of pelvic pain and dyspareunia. Coronal (**a**) and axial (**b**) enhanced CT images. Multinodular, heterogeneous and bilateral ovarian tumour, with moderate enhancement after contrast administration. Ascites are found in the pelvis and parieto-colic gutters. Multiple liver metastases were also seen (not shown)

We review three cases of neuroendocrine ovarian tumours, one primary carcinoid and two secondary carcinoid tumours (Figs. 1, 2 and 3). Once again, we emphasise that few data are provided in the literature regarding the imaging features of these tumours. However, globally our findings match the literature concepts. All the cases show well-circumscribed, predominately solid ovarian tumours. However, Fig. 1 exemplifies a typical benign behaviour, while Fig. 3 shows several imaging criteria of malignant, metastatic disease. Figure 2 represents an atypical presentation of metastatic disease, with overlap between benign and malignant features.

## Cervix

The most common NE cervical tumour is small cell carcinoma. Large cell NE carcinoma and carcinoid types are rarer than the former and few data can be found in the literature.

## Poorly differentiated neuroendocrine carcinomas (NECs)

As previously established, NECs encompass small cell carcinoma and large cell NE carcinoma. Staging and treatment strategies for both small and large cell cervical carcinoma globally follow the same criteria used for common histological types of cervical carcinomas [1].

Small cell carcinoma accounts for 1–6 % of cervical carcinomas [6, 27]. Median age of diagnosis is in the 5th decade, ranging from 21 to 87 years. Its clinical presentation is non-specific, typically including vaginal bleeding. Some patients present with a cervical mass and an abnormal Pap smear may be found. This tumour is strongly associated with human papilloma virus 18 (HPV-18), but HPV-16-positive tumours have also been demonstrated [28].

Rarely, paraneoplastic manifestations due to ectopic hormone production may appear. The most commonly found are hypercalcaemia (parathormone), hypoglycaemia (insulin), carcinoid syndrome (serotonin), Cushing’s syndrome (corticotropin), SIADH (vasopressin) and myasthenia gravis [29].

Most tumours are voluminous, exceeding 6 cm, and show deep cervical infiltration and necrosis. Often, growth may be exophytic, which appears to be associated with a better prognosis [6]. Nevertheless, it is generally a highly aggressive tumour with a worse prognosis than that of poorly differentiated squamous cell carcinomas. The rates of lymphovascular space invasion and extra-pelvic recurrences for bone, supraclavicular lymph nodes and lung are particularly high [6, 27, 28, 30]. According to McCusker et al., females with small cell tumours have 1.84 times greater risk of death compared to patients with squamous cell carcinomas [31].

Large cell neuroendocrine carcinoma of the cervix is a rare, aggressive tumour. It appears to have a similar outcome to small cell carcinoma and its treatment remains challenging because of the high rate of recurrence and distant metastases even with early stage disease [2, 32]. The most frequent metastatic sites include the central nervous system, lung and bone [33].

**Fig. 4:**
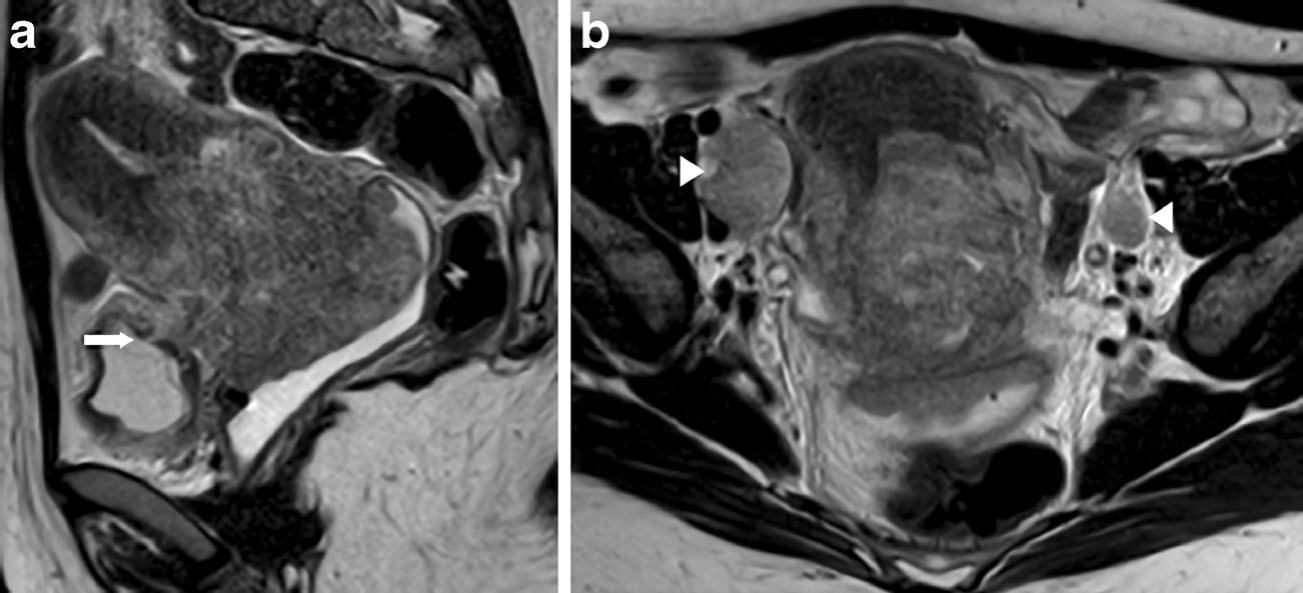
**a**–**b** Cervical neuroendocrine tumour (FIGO IV-A; invasion of adjacent organs). A 32-year-old female complaining of vaginal bleeding after intercourse and fetid vaginal discharge. Sagittal T2-weighted MR image (**a**) and axial T2-weighted MR image (**b**). Large cervical cancer with infiltration of the lower segment of the uterine corpus. The parametria are circumferentially infiltrated and the tumour extends anteriorly to the bladder, with transmural invasion and fistulae (*arrow*). Bilateral, nodal metastases of the external iliac groups are also seen (*arrowheads*)

**Fig. 5:**
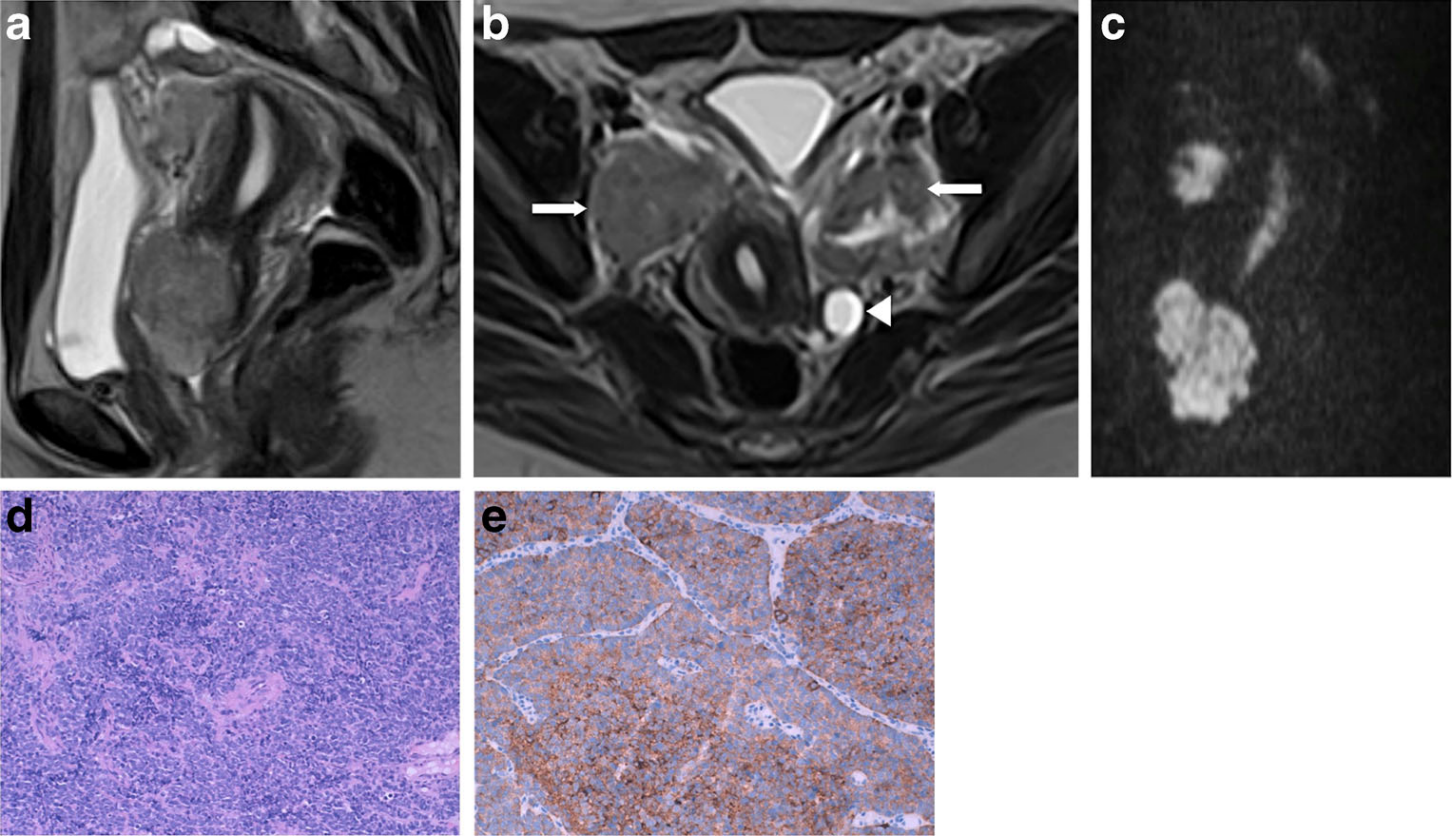
**a**–**e** Cervical small cell carcinoma (FIGO II-A; invasion of the upper two-thirds of vagina with no parametrial invasion). A 41-year-old female complaining of vaginal bleeding after intercourse. Sagittal T2-weighted MR image (**a**), axial T2-weighted MR images (**b**) and sagittal diffusion weighted image (DWI), *b*–(**c**) Retroverted uterus with an anterior cervical tumour, highly restrictive on DWI. No parametrial invasion is noted, but huge, bilateral obturator lymph nodes are found (*arrows*). The left ovary shows follicular activity and is posteriorly displaced (*arrowhead*). In this H&E section the cervix is occupied by a solid tumour, composed of small cells, with uniform nuclei and scarce cytoplasm (**d**). Some tumour cells show positive cytoplasmatic staining for synaptophysin (**e**) and for chromogranin (not shown)

**Fig. 6:**
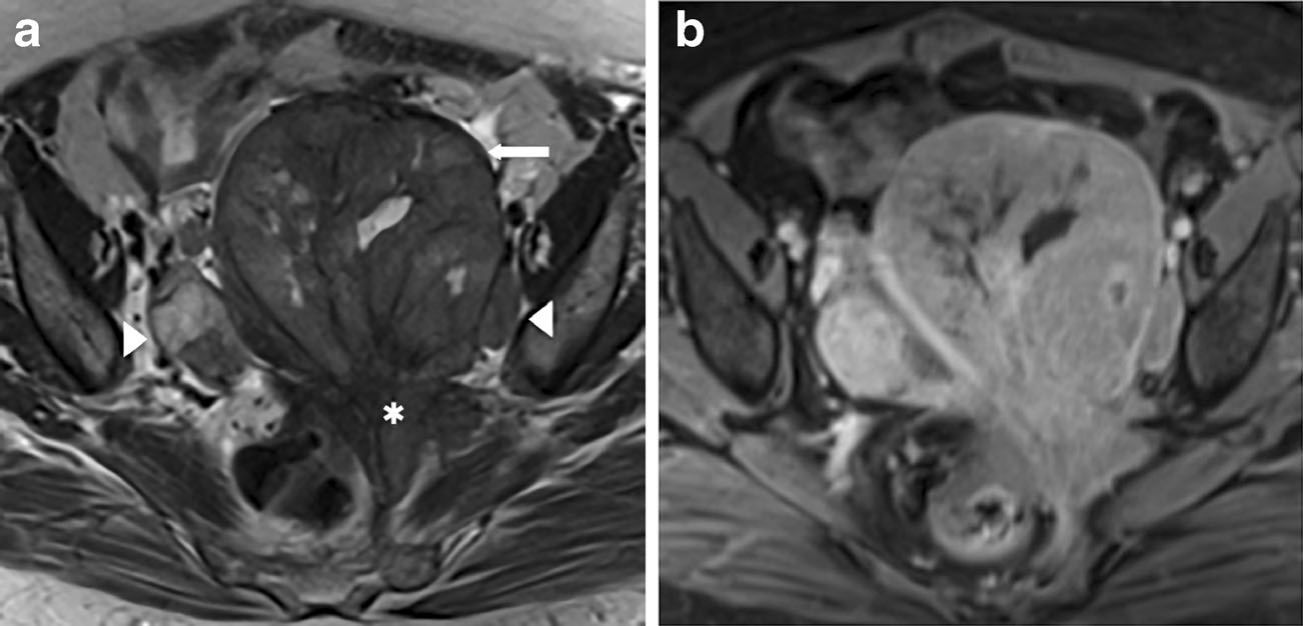
**a**–**b** Cervical small cell carcinoma (FIGO III-B; extension to the pelvic side wall). A 64 year-old female complaining of menorrhagia. Axial T2-weighted MR image (**a**) and axial T1-weighted MR image with fat saturation after intravenous gadolinium administration (**b**). Cervical tumour extending posteriorly to the pelvic wall (*asterisk*) and superiorly to the left adnexal area, where it forms a huge solid, heterogeneous mass with central necrotic areas (*arrow*). This mass shows intense enhancement after gadolinium administration and is adherent to the uterine corpus, which may be found inferiorly. Bilateral internal iliac lymph nodes are also noted (*arrowheads*)

We review three cases of cervical small cell carcinoma (Figs. 4, 5 and 6). Cervical NECs resemble cervical squamous cell carcinoma, displaying the same MRI features: low signal intensity on T1-weighted images (T1WI) and high signal intensity on T2-weighted images (T2WI). No histological distinction can be provided regarding only imaging features. However, according to the literature, cervical NECs are generally highly aggressive and expected to be diagnosed in advanced FIGO stages. Concordantly, we illustrate NECs in IV-A (invasion of adjacent organs) (Fig. 4) and III-B (invasion of the pelvic side wall) (Fig. 6) stages.

## Well-differentiated neuroendocrine tumours (NETs)

Well-differentiated neuroendocrine tumours (NETs) include typical and atypical carcinoid tumours. Among the uncommon sites of primary carcinoid tumours, the uterine cervix constitutes one of the rarest locations, representing only 0.5 % to 5 % of cervical cancers. The most common symptom is also vaginal bleeding. Carcinoid syndrome is very rare and even in the lack of clinically evident carcinoid syndrome, many patients present with elevated urinary 5-hydroxyindoleacetic acid (5-HIAA). However, the diagnosis of a cervical carcinoid is frequently postoperative and laboratorial investigation is typically not helpful since it is not performed in the absence of symptoms. These tumours display a very aggressive, malignant behaviour, and their prognosis is poor (2- or 3-year survival rates, ranging from 12.5 to 33 %, according to some studies). This emphasises the importance of early diagnosis and treatment [14].

Atypical carcinoids of the cervix are also very rare and, according to a review of Yoshida et al. [34], only 13 cases have been reported in the literature.

## Endometrium

The reported endometrial neoplasms with NE differentiation essentially include small cell carcinoma and endometrial adenocarcinoma with NE cells [5].

Small cell carcinoma of the endometrium resembles that of the lung and is uncommon, accounting for less than 1 % of all endometrial carcinomas. Nonetheless, more than 50 cases have been reported in the literature. The age of diagnosis ranges from 23 to 78 years old. Like in other endometrial carcinomas, abnormal bleeding is the primary presenting symptom, but some patients present with advanced disease, sometimes with pain from metastases [5]. These tumours usually present as bulky intraluminal masses, and deep myometrial invasion (33–50 %) and extra-uterine spread (near 50 %) are common. Pelvic lymph nodes, adnexa and the peritoneum constitute the typical sites of extra-uterine involvement. The overall prognosis is poor [5, 35].

In 50 to 66 %, endometrial small cell carcinoma displays an admixed component of grade 1 or 2 endometrioid adenocarcinoma, and few cases showed a malignant mixed mullerian tumour within a predominant small cell carcinoma [5].

**Fig. 7:**
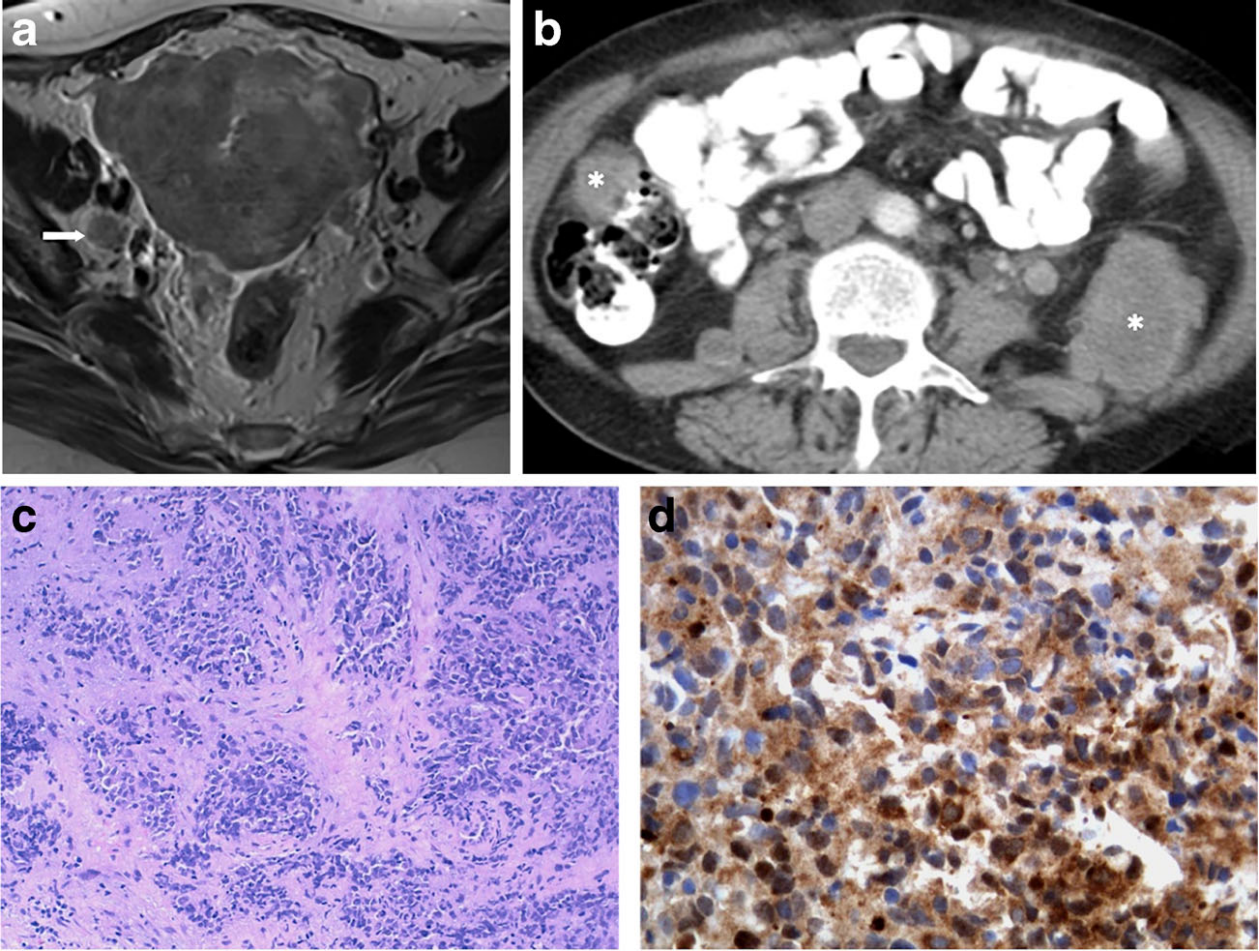
**a**–**d** Poorly differentiated endometrial carcinoma with NE differentiation (FIGO IV-B; distant metastases). A 61-year-old female complaining of metrorrhagia. Axial T2-weighted MR image (**a**) and axial enhanced CT image (**b**). Large endometrial tumour invading the myometrium, serosa and surrounding adipose tissues. There is no invasion of the cervical stroma or bladder (not shown), but a right obturator node (*arrow*) is found. CT evaluation shows that the disease has disseminated outside the pelvis, revealing para-aortic lymph nodes (*arrowhead*) as well as retroperitoneal and peritoneal metastases (*asterisks*). Vagina biopsy (H&E) infiltrated by a solid tumour composed by sheets of uniform cells, with small oval nuclei and scarse cytoplasm (**c**). These cells have an intense cytoplasmatic staining for neuron-specific enolase (**d**) and have small chromogranine-positive granules in the cytoplasm (not shown). This tumour was synaptophysin negative

**Fig. 8:**
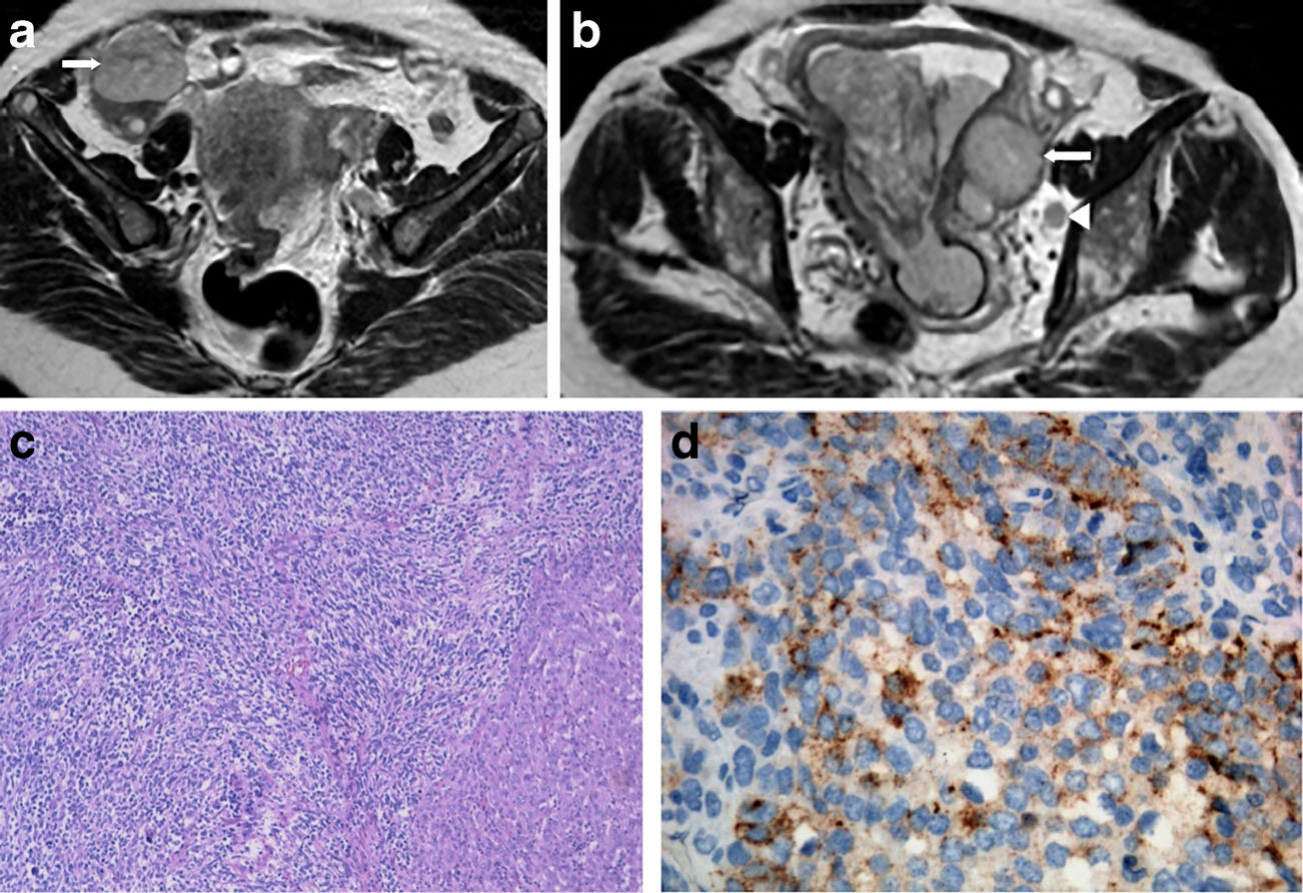
**a**–**d** Undifferentiated endometrial carcinoma with NE differentiation (FIGO III-C1; involvement of pelvic nodes). A 67-year-old female complaining of metrorrhagia. Axial T2-weighted MR images (**a**, **b**). Endometrial tumour prolapsing into the cervical canal, without invasion of the cervical stroma and myometrium. The tumour extends bilaterally through the lumen of the tubes to the parametrial tissues (*arrows*). A left obturator lymph node is also seen (*arrowhead*). H&E section of the tumour with a solid pattern composed by large cells with pleomorphic nuclei, mitoses and scarce cytoplasm (**c**). These cells have small chromogranine-positive granules (**d**) and small synaptophysin granules in the cytoplasm (not shown) and are strongly positive for pancytokeratin CAM 5.2 (not shown)

We review two cases of poorly differentiated endometrial carcinoma with NE differentiation (Figs. 7 and 8). Like in cervical carcinoma, imaging does not allow histological distinction, since all endometrial cancers tend to present similarly: low to moderate signal on T2WI and hypointensity relative to the hyperintense enhancing myometrium on dynamic contrast-enhanced MRI sequences. However, when a highly aggressive endometrial tumour is found, a neuroendocrine nature may be suspected. There are no consistent data regarding the prognosis of non-pure NE endometrial tumours; however, the cases we illustrate were diagnosed in advanced FIGO stages, IV-B (distant metastases) (Fig. 7) and III-C1 (involvement of pelvic nodes) (Fig. 8).

## Other locations

Neuroendocrine tumours of the fallopian tube, vagina and vulva are very rare and only a few cases have been reported [5].

In the fallopian tube, these tumours encompass carcinoids and small cell carcinoma, and they are not well known. In the vagina, almost ten cases of small cell carcinoma have been reported, occurring in females between 41 and 78 years of age. Like in other vaginal tumours, the most frequent complaint was vaginal bleeding or discharge. Metastatic disease was common in this small cohort, so the prognosis is thought to be poor. In the vulva, neuroendocrine differentiation may be found in small cell carcinoma of the Bartholin gland and Merkel cell tumours, which tend to present as vulvar masses [5].

## Conclusion and teaching points

NE tumours of the female genital tract are rare, either primary or secondary. Two groups are currently considered: NECs, including small cell carcinoma and large cell NE carcinoma, and NETs, which encompass typical and atypical carcinoids.

The clinical features of theses tumours are non-specific and depend on the organ of origin and on the extension and aggressiveness of the disease. The imaging evaluation should follow the same criteria used for other tumours, and FIGO remains the preferred staging system. The diagnosis is obviously histological, and the role of imaging tools essentially includes initial detection, staging and biopsy guidance. However, some points should be taken into account when imaging studies are performed:

- Cervical small cell carcinoma and ovarian carcinoid are the most common gynaecological NE tumours.
- Cervical small cell carcinomas usually behave aggressively.
- Ovarian carcinoids tend to behave in a benign fashion.
- While managing an ovarian carcinoid, extra-ovarian extension, bilaterality and multinodularity should raise the suspicion of metastatic disease.
- Endometrial NE tumours are very rare and are usually small cell carcinoma or adenocarcinoma with NE differentiation.
- As for other tumours, US, CT, PET-CT and MRI remain the most commonly used imaging tools, but octreotide scans may be useful for typical and atypical carcinoids.

## Abbreviations

NE: neuroendocrine
NECs: poorly differentiated neuroendocrine carcinomas
NETs: well-differentiated neuroendocrine tumours
MEEC: mixed exocrine-endocrine carcinoma
WHO: World Health Organisation
SCC: squamous cell carcinoma
FIGO: International Federation of Gynaecology and Obstetrics
US: ultrasound
CT: computed tomography
PET-CT: positron emission tomography-computed tomography
MRI: magnetic resonance imaging
HPV: human papilloma virus
T1WI: T1-weighted imaging
T2WI: T2-weighted imaging
