# Erratum to: Neuroendocrine tumours of the female genital tract: a case-based imaging review with pathological correlation

**DOI:** 10.1007/s13244-015-0400-6

**Published:** 2015-03-24

**Authors:** João Lopes Dias, Teresa Margarida Cunha, Filipe Veloso Gomes, Catarina Callé, Ana Félix

**Affiliations:** 1 Department of Radiology, Hospital de São José, Centro Hospitalar de Lisboa Central, Lisbon, Portugal; 2 Department of Radiology, Instituto Português de Oncologia de Lisboa, Francisco Gentil, Lisbon, Portugal; 3 Department of Radiology, Centro Hospitalar do Algarve, Faro, Portugal; 4 Department of Pathology, Instituto Português de Oncologia de Lisboa, Francisco Gentil, Lisbon, Portugal; 5 Department of Pathology, Instituto Português de Oncologia de Lisboa, Francisco Gentil, Lisbon, Portugal

**Erratum to: Insights Imaging**

**DOI 10.1007/s13244-014-0378-5**

Due to a transcription error, the authors’ affiliations were incorrectly published. We would like to clarify that the authors’ affiliations should read as listed below. We apologise for this error.

We would also like to thank Manuel Abecasis, MD, Tiago Saldanha, MD, Adalgisa Guerra, MD, and Hugo Pisco Pacheco, MD, for providing us with MR images of Figures 2, 5, 6 and 7, respectively.

